# Comparative Transcriptome Analysis of Hepatopancreas Reveals Sexual Dimorphic Response to Methyl Farnesoate Injection in *Litopenaeus vannamei*

**DOI:** 10.3390/ijms25158152

**Published:** 2024-07-26

**Authors:** Zhihui Yang, Xiaoliu Yang, Jiahao Du, Cun Wei, Pingping Liu, Jingjie Hu, Zhenmin Bao, Zhe Qu

**Affiliations:** 1 MOE Key Laboratory of Marine Genetics and Breeding, Ocean University of China, Qingdao 266003, China; meswyzh@163.com (Z.Y.);; 2 Key Laboratory of Tropical Aquatic Germplasm of Hainan Province, Sanya Oceanographic Institution, Ocean University of China, Sanya 572024, Chinasoiweic@ouc.edu.cn (C.W.); 3 Southern Marine Science and Engineering Guangdong Laboratory (Guangzhou), Guangzhou 511458, China

**Keywords:** *Litopenaeus vannamei*, methyl farnesoate, sex dimorphism, transcriptome, hormonal regulation

## Abstract

Sexually dimorphic traits such as growth and body size are often found in various crustaceans. Methyl farnesoate (MF), the main active form of sesquiterpenoid hormone in crustaceans, plays vital roles in the regulation of their molting and reproduction. However, understanding on the sex differences in their hormonal regulation is limited. Here, we carried out a comprehensive investigation on sexual dimorphic responses to MF in the hepatopancreas of the most dominant aquacultural crustacean—the white-leg shrimp (*Litopenaeus vannamei*). Through comparative transcriptomic analysis of the main MF target tissue (hepatopancreas) from both female and male *L. vannamei*, two sets of sex-specific and four sets of sex–dose-specific differentially expressed transcripts (DETs) were identified after different doses of MF injection. Functional analysis of DETs showed that the male-specific DETs were mainly related to sugar and lipid metabolism, of which multiple chitinases were significantly up-regulated. In contrast, the female-specific DETs were mainly related to miRNA processing and immune responses. Further co-expression network analysis revealed 8 sex-specific response modules and 55 key regulatory transcripts, of which several key transcripts of genes related to energy metabolism and immune responses were identified, such as arginine kinase, tropomyosin, elongation of very long chain fatty acids protein 6, thioredoxin reductase, cysteine dioxygenase, lysosomal acid lipase, estradiol 17-beta-dehydrogenase 8, and sodium/potassium-transporting ATPase subunit alpha. Altogether, our study demonstrates the sex differences in the hormonal regulatory networks of *L. vannamei*, providing new insights into the molecular basis of MF regulatory mechanisms and sex dimorphism in prawn aquaculture.

## 1. Introduction

Juvenile hormone (JH) and methyl farnesoate (MF) are well-known sesquiterpenoid hormones that play important roles in arthropod growth and reproduction [1]. As a structurally similar analogue of insect JH, MF is secreted from the mandibular organ of various crustaceans and plays crucial roles in regulating their growth, molting, reproduction, and behavior [1,2]. The synthesis and degradation of these sesquiterpenoid hormones are conserved in insects and crustaceans, except for the lack of enzymes producing JH in crustaceans [1,3]. The carboxylesterases are responsible for their ester hydrolysis, and JH esterase-like carboxylesterase (*JHE-L*) has been characterized in several crustaceans [4,5]. The JH receptor (*Met*) and downstream responsive genes such as *Kr-h1* have also been characterized in various crustaceans [6,7]. Similar to insect JH, crustacean MF could use Met as its hormone-responsive receptor for downstream regulations to function in target tissues [8,9]. The study in the swimming crab *Portunus trituberculatus* demonstrated that MF induced *Met* expression in both the hepatopancreas and ovary in vivo [8]. It has also been revealed that MF binds to crustacean Met that dimerizes with the protein steroid receptor coactivator (SRC) to activate downstream gene transcription [10].

The molting process is crucial to crustacean growth, and the influence of MF on molting has been reported in different crustacean species [11,12,13]. For example, MF could directly stimulate the secretion of ecdysteroids in *Cancer mgister* Y-organs [13]. Both MF and ecdysone pathways are involved in the precise regulation of molting, and the crosstalk between these two hormone pathways was discussed [14]. Recently, a comparative transcriptomic analysis has been conducted in female *Macrobrachium nipponense* after 20-hydroxyecdysone injection, revealing the relevant molecular basis of hormonal regulation and molting [15]. In contrast, little was known about the transcriptome response to MF. During crustacean reproduction, the female is characterized by the rapid synthesis of vitellogenin contributing to gonad maturation [16]. Transcriptome, proteome analyses, and in situ expression experiments have indicated that the hepatopancreas and ovary are the main production and accumulation tissues of vitellogenin during female reproduction [17]. The important roles of MF in stimulating gonadal maturation and vitellogenesis are well established [1,17,18]. Also, increasing evidence for MF regulation on vitellogenesis in various crustaceans suggests conserved mechanisms exist in crustaceans and insects [9]. *Daphnia pulex* is a crustacean with a unique reproductive system producing female offspring asexually or male sexually in response to different environmental cues, and MF is necessary for the production of male offspring during sexual reproduction [19,20].

In addition, sesquiterpenoid hormones were also found to be involved in the formation of sexual dimorphism. In insects, JH plays a crucial role in regulating gender development and phenotype formation. Studies have shown that *doublesex* regulates sex-specific mandible growth via JH signaling, and the Fat/Hippo signaling pathway coordinates the link between the whole-body JH signal and tissue-specific developmental pathways in stag beetles [21,22]. Sexually dimorphic traits are also widespread in crustaceans, and clues to the link between MF and phenotype have been reported. In prawn, the different titers of MF in the hemolymph of males and females were found [23]. However, little is known about the differences in MF regulation between male and female crustaceans, and the molecular mechanisms underlying the regulation of MF on sexual dimorphism require more research. Thus, further exploration of the different molecular responses of both sexes to MF will help gain a more comprehensive understanding on the mechanism of sexual dimorphism in crustaceans.

*Litopenaeus vannamei*, also known as the white-leg shrimp, is one of the most popular species in the aquaculture industry due to its high yield, fast growth rate, and easy adaptability to different environmental conditions. The production of *L. vannamei* reached 5.8 million tons in 2020, which accounted for 51.7% of total crustacean production in the world representing the most economically important marine crustaceans farmed [24]. *L. vannamei* often exhibits various sexually dimorphic traits, such as different feeding behaviors, and females are superior to males in growth rate, body size, and weight [25,26]. However, the sex dimorphic hormonal regulation of *L. vannamei* remains unknown. Thus, studying white-leg shrimp *L. vannamei* comprises both practical and scientific importance. To gain a comprehensive understanding of MF regulation on the sexually dimorphic traits of *L. vannamei*, elucidating the underlying differences between the responses of both sexes is crucial. Here, we conducted a systemic investigation on the transcriptomic responses of the main target tissue of *L. vannamei* to sesquiterpenoid hormone-MF, and revealed the sexual dimorphic responses and hormonal regulatory networks behind.

## 2. Results

### 2.1. Overview of Hepatopancreases Transcriptome Data after Injection

In this study, a total of 18 RNA-Seq libraries of the hepatopancreases from shrimps of both sexes were conducted and sequenced. A total of 1,145,354,794 clean reads with high quality were obtained with an average of 63,630,821 for each sample after filtering. The mapping rates of the samples were all above 93.23%. The detailed information for the sequencing data was listed in Supplementary information Table S1. In our transcriptomic analysis, a total of 60,412 transcripts belonging to 25,300 unigenes were obtained, of which 9.87% were transcription factors (TF) and 61.03% TF had variant transcripts.

### 2.2. Transcriptomic Responses of Hepatopancreases to MF Injection in L. vannamei

**Figure 1:**
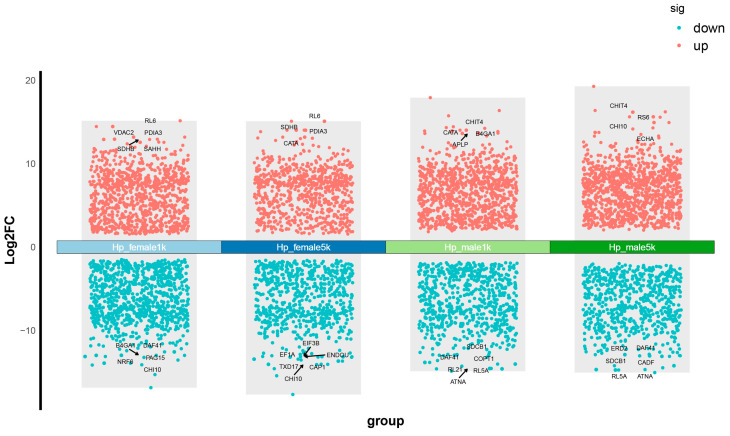
Composite volcano plot showing the DETs in hepatopancreases of different MF injection groups compared to their own control group. The red and green dots represent the up- and the down-regulated transcripts with |Log2FC| > 1.5 and *p*-value < 0.05. The top 10 annotated DETs based on |Log2FC| values are labeled.

The DETs between each dosage and relevant control of the female and male groups were identified separately. There were 769–1122 up-regulated and 648–885 down-regulated transcripts identified among the comparisons (Figure 1, Figure S1A). Additionally, there were 19 and 12 commonly up-regulated and down-regulated DETs in all groups, respectively (Figure S1B). The expression of the DETs between the female and male groups at the gene level was also investigated. There were 583 common differentially expressed genes (DEGs) between the female and male groups, which included 1336 DETs. There were 3443 sex-specific DEGs, including 3754 DETs. Interestingly, for the common DEGs, as many as 65.87% had splicing variants with differential expression. However, for sex-specific DEGs, only 7.32% had splicing variants with differential expression. Transcription factors were further analyzed, and 12.86% of the common DEGs are TF, of which as much as 73.33% have splicing variants with differential expression. Among the sex-specific DEGs, 11.01% are TF, while only 10.63% of these TF had differentially expressed splicing variants.

### 2.3. Sexually Dimorphic Responses of Key Components in Hormone-Related Pathways

To examine the sexual dimorphic responses of genes in hormone-related pathways, the expression changes in the key factors involved in MF and ecdysone hormonal pathways such as *JHE-L*, *Met*, *Kr-h1*, *Broad*, *ECR*, *E75*, *HR3*, *Ftz-F1*, *Vg*, and *VgR* were analyzed (Table S2). For the *JHE-L*, three transcripts were found to be up-regulated, while another four were found to be down-regulated in both sexes. Interestingly, after high-dose MF injection, one transcript of *JHE-L* was specifically up-regulated in the males, but down-regulated in the females. For the MF receptor *Met*, two splicing variants (Table S2) were specially up-regulated in female and male shrimps, respectively. The MF-targeted transcription factor *Kr-h1* and response gene *Broad* exhibited opposite regulatory trends in female and male shrimps. For the ecdysone receptors, *ECR* were found to be down-regulated in both sexes. Two ecdysone-responsive nuclear receptors (*E75*, *HR3*) were down-regulated in both sexes. In addition, one transcript of *Ftz-F1* was up-regulated and another two were down-regulated in the females, while none of these transcripts were differentially expressed in the males. Regarding vitellogenesis, although only a low expression level of *Vg* was detected, two splicing variants of *VgR* were specifically up-regulated in males.

### 2.4. Clusters of the Gender-Specific Responding DETs

**Figure 2:**
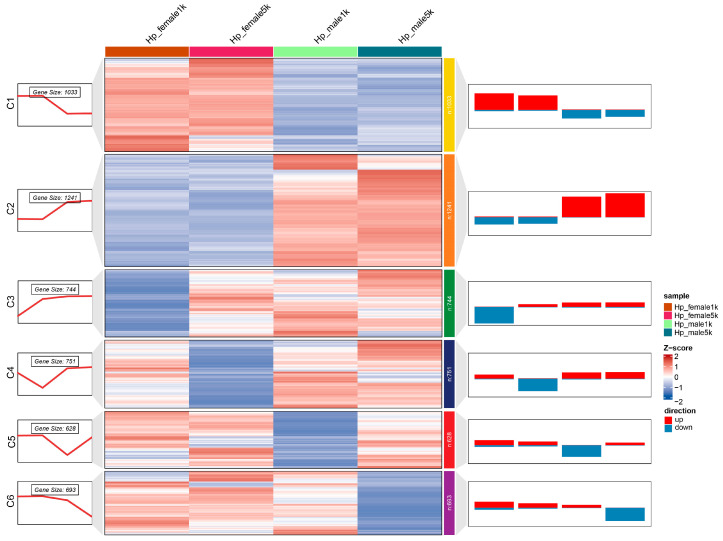
The clustering result of DETs profile determined by Log2FC values. The right bar charts show the numbers of the up- (red) and down-regulated (blue) DETs of different groups in corresponding clusters. The left line charts show the average value of Log2FC values in clusters. The heatmap represents the expression profile determined by Log2FC values, and the hierarchical clustering was performed with the “mfuzz” method in R package ClusterGVis [27]. The numbers in the color blocks represent the number of transcripts in each cluster. C1–C6, the six clusters of DETs.

The DETs were clustered into six clades (C1–C6) and exhibited the sex- and dose-specific patterns (Figure 2, Table S3). For the sex-specific clusters, the 708 DETs of the C1 cluster were up-regulated significantly in the female groups (Hp_female1k, Hp_female5k), while the 1013 DETs of the C2 cluster were specifically up-regulated in the male groups (Hp_male1k, Hp_male5k). Moreover, the dose-specific down-regulation of DETs was found in both sex groups (C3, C4 for female; C5, C6 for male). Among them, 490 DETs of the C3 cluster were specifically down-regulated in the low-dose female group, 372 DETs of the C4 cluster were specifically down-regulated in the high-dose female group, 350 DETs of the C5 cluster were specifically down-regulated in the low-dose male group, and 386 DETs of the C6 cluster were specifically down-regulated in the high-dose male group (Figure 2).

### 2.5. GO and KEGG Enrichment Analysis of the Gender-Specific Responding DETs Clusters

To investigate the potential regulatory functions of the DETs, GO and KEGG enrichment analysis was performed. A total of 2231 GO terms were significantly enriched (*p* < 0.05, Supplementary Table S4). For the female-specific up-regulated DETs (C1 cluster), the main enriched GO terms were related to the miRNA processing of the biological process category, the “oxidoreductase complex”, “pronucleus”, and “Dsl1/NZR complex” of the cellular component category, and “5S rRNA binding”, “nucleosome binding”, and “NAD binding” of the molecular function category. For the male-specific up-regulated DETs (C2 cluster), they were mainly assigned to sugar metabolism in the biological process category, such as the “oligosaccharide metabolic process”, “amino sugar metabolic process”, “chitin catabolic process”, and “chitin metabolic process”. In the molecular function category, the “chitinase activity”, “beta-fructofuranosidase activity”, and “sucrose alpha-glucosidase activity” were highly represented. We further list the details of the chitinase DETs from C2 in Table S5. For the female–dose-specific DETs (C3 and C4 cluster), the enriched GO terms were mainly related to collagen and fatty acid metabolism and cellular homeostasis, the vesicle and RNA polymerase complex, carrier activity, and transcription factor activity. For the male–dose-specific DETs (C5 and C6 cluster), the DETs of the C5 cluster were enriched in the “chromosome condensation”, “regulation of carbohydrate metabolic process”, and “sarcosine metabolic process” of the biological process category, “chromaffin granule” and “nucleosome” of the cellular component category, and methyltransferase activity of the molecular function category. Meanwhile, the C6 cluster was mainly enriched in galactolipid metabolism and amino acid biosynthesis.

**Figure 3:**
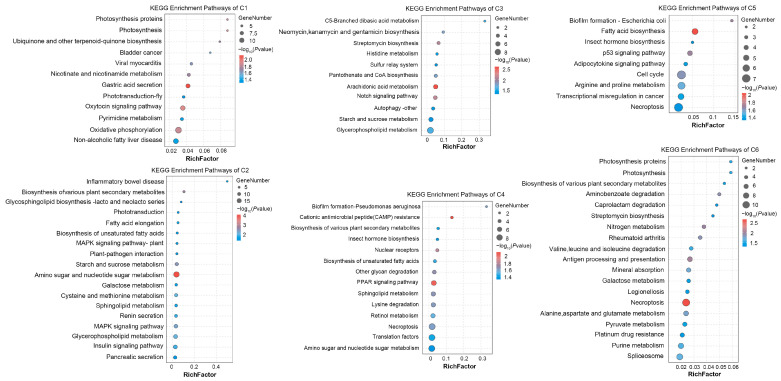
KEGG pathway enrichment of DETs in C1–C6 clusters.

Furthermore, these DETs were enriched in different KEGG pathways. A total of 12, 18, 11, 14, 9, and 19 pathways were significantly enriched for the DETs of the C1–C6 clusters, respectively. The enriched KEGG pathways of each cluster were displayed in Figure 3. Most of the enriched pathways were related to diseases and immunity, sugar and lipid metabolism, and related signal pathway regulation. For the disease- and immunity-related pathways, “Bladder cancer”, “Non-alcoholic fatty liver disease”, “CAMP resistance”, and “Platinum drug resistance” were identified. For the sugar metabolism pathways, “Amino sugar and nucleotide sugar metabolism” related to chitin metabolism was represented, and “Starch and sucrose metabolism” and “Galactose metabolism” were included. For the lipid metabolism, “Glycerophospholipid metabolism”, “Sphingolipid metabolism”, “Glycerolipid metabolism”, “Biosynthesis of unsaturated fatty acids”, “Fatty acid biosynthesis”, and “Fatty acid elongation” were included. For the signal pathways, the “p53 signaling pathway”, “PPAR signaling pathway”, “Notch signaling pathway”, “Adipocytokine signaling pathway”, “Insulin signaling pathway”, and “MAPK signaling pathway” were identified.

### 2.6. Sexual Dimorphic Modules Responding to Sesquiterpenoid Hormone

**Figure 4:**
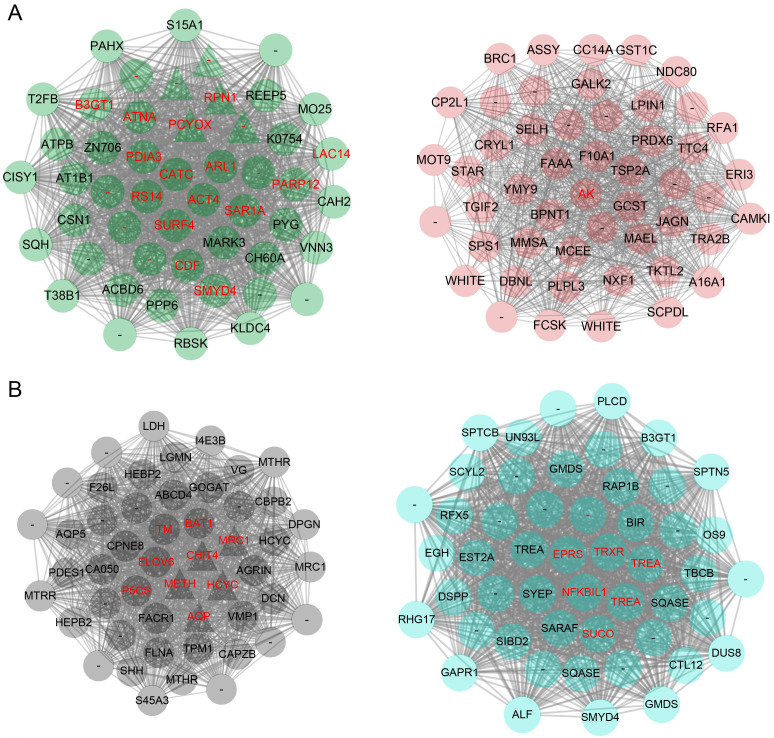

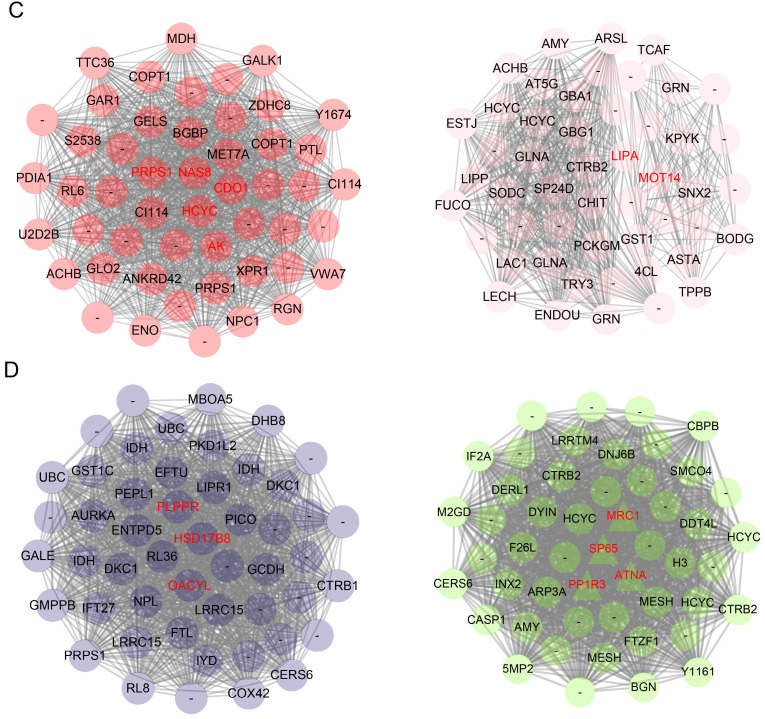
Sexual dimorphic co-expression modules responding to sesquiterpenoid hormone. (**A**) Co-expression networks of the female-specific responding modules. (**B**) Co-expression networks of the male-specific responding modules. (**C**) Co-expression networks of the female–dose-specific responding modules. (**D**) Co-expression networks of the male–dose-specific responding modules. Circle color corresponds to module color. Triangles represent male-specific transcripts. The annotated transcripts were labeled, while the ones without annotation were labeled with “-”. The key DETs in the module were marked in red.

With the purpose of exploring key networks and the specific hub transcripts of sexual dimorphic responses to the MF hormone, the WGCNA was performed. A total of 15,080 annotated transcripts (TPM > 0.1, top 25% by variance) were selected for the co-expression network construction. The soft threshold was selected as 10, and the minimum module size was set to 100. Similar modules were merged with a threshold of 0.25, and 18 co-expression modules were obtained (Figure S2A). The module eigengenes were calculated to represent each module and the relationships among the identified modules were displayed using the hierarchical clustering tree (Figure S2B) and adjacency heatmap (Figure S2C). The results indicated that the co-expressions of transcripts were highly independent between modules. The significance of the sex-specific DETs enriched in each module was analyzed to identify the corresponding sex-specific modules in response to MF (Figure S3). In each module, the top 50 transcripts with the highest connectivity were identified as hub transcripts. The hub transcripts appearing in both sex-specific modules and corresponding DET clusters were considered to be the key transcripts. Finally, a total of eight key modules including two male-specific, two female-specific, two male–dose-specific, and two female–dose-specific modules responding to MF were identified (Figure 4). And a total of 55 key transcripts in 8 key modules were identified for the sexual dimorphic responses (Figure 4), and the details are listed in Table S6.

## 3. Discussion

Sexual dimorphism is widely found in crustaceans, and the differences of growth-related commercial traits such as body weight and size are of great concern to the aquaculture industry. During the culture of *L. vannamei*, the females usually gain a bigger body size over the males. The growth of crustaceans is accompanied by the replacement of the chitinous exoskeleton during the molting process, which is a complex process regulated by multiple factors such as hormones, photoperiod, and nutritional conditions [28]. MF is a well-known sesquiterpenoid hormone playing key roles in crustacean growth and reproduction. Much research was conducted to explore the functions of MF in shrimps [8,9,10,16]. However, little is known about the sexually dimorphic responses of shrimps to MF. In crustaceans, the hepatopancreas is closely related to processes such as molting, gonadal development, metabolism, and immunity [29]. As one of the key effector organs of the sesquiterpenoid hormone MF, the hepatopancreas has been reported to be involved in decapod molting and female gonad maturation [17,18,30]. In this study, a comprehensive transcriptome analysis was performed in the hepatopancreas to understand the differences between the female and male *L. vannamei* responding to MF injection.

We first observed the expression changes in the key genes related to hormonal pathways after MF injection. The juvenile hormone esterase-like carboxylesterase JHE-L is the key enzyme in MF degradation, playing a vital role in MF titer regulation [5]. In *L. vannamei*, *JHE-L* was found to have undergone gene expansion and was highly expressed in the hepatopancreas [5]. In our study, multiple transcripts of *JHE-L* were up-regulated after MF injection. Similar increases in the expression of *JHE-L* were observed in other crustaceans treated with MF [31,32]. As the MF has been reported in the regulation of molting and reproduction including vitellogenesis, we also looked into the expression of the genes involved in related pathways. The *Met* gene has been identified in various crustaceans, and research has shown the regulatory role of MF on the expression of the *Met*, as well as their interaction [10,33]. *Met* is increasingly recognized as the receptor for the MF hormone in crustaceans, and we found two splicing variants of *Met* were up-regulated after MF injection, indicating the regulation of MF on *Met* in *L. vannamei*. Interestingly, these two transcripts showed sex bias, suggesting a dimorphism in the transcriptional response of female and male shrimp to MF. *Kr-h1* and *Broad* is characterized as the targeted transcription factor and response gene of sesquiterpenoid hormones in insects [34,35]. In this study, the differential expression of *Kr-h1* and *Broad* was found only in male shrimps after MF injection. And down-regulated *Kr-h1* and up-regulated *Broad* were observed, which was the opposite with insects [14]. In addition, a study on mud crab *Scylla paramamosain* showed no associated response to MF in *Kr-h1* expression [33]. Thus, although these two genes have been identified in various crustaceans, their functional roles need to be further elucidated. For the ecdysone pathway, the ecdysone receptor *ECR* and response gene *E75*, *HR3* were down-regulated in both sexes of *L. vannamei*, indicating the antagonism between MF and the ecdysone pathway. Moreover, the differential expression of the response gene *Ftz-F1* was observed only in female shrimps with different splicing variant regulation, further suggesting a sexually dimorphic response to hormone administration.

In our comparative transcriptome analysis, a large number of common DEGs were found between male and female shrimps after MF injection, but only a small proportion of DETs were regulated commonly in both sexes. Further analysis showed that the most common DEGs possessed multiple splicing variants, and different variants were differentially regulated in female and male shrimps. Our results indicated the significant differences of female and male shrimps responding to MF at the splicing variants’ level. And different isoforms were chosen responding to MF injection. For female individuals, the specifically up-regulated DETs clustered in C1 were enriched in miRNA biogenesis-related terms with high significance, of which several genes such as DEAD-box helicase 17 (*DDX17*) and mothers against decapentaplegic homolog 3 (*SMAD3*) attracted our special attention. DDX17 is an ATP-dependent RNA helicase from the large family of DEAD-box RNA helicases. In mammals, DDX17 is a multifunctional helicase important for various contexts, including the processing of pri-miRNA, pre-mRNA alternative splicing, RNA remodeling, coregulation of transcription, and immune response [36]. However, studies of DDX17 in crustaceans are still lacking. Additionally, the SMAD protein is a cofactor which can be recruited by DEAD-box proteins for the positive regulation of miRNA processing [37]. In crustaceans, studies indicated that SMAD3 played roles in immune responses [38,39,40]. And the up-regulation of SMAD3 could inhibit apoptosis in the hepatopancreas of *L. vannamei* [40]. The enrichment of DETs in miRNA processing indicated the importance of miRNA regulation in responses to MF injection, and the screening of related miRNA as well as their interaction with targets, such as immune-related genes, deserves further studies. Additionally, the oxidative phosphorylation pathway, the primary source of cellular energy in eukaryotes [41], was also enriched in our result. A similar up-regulation of oxidative phosphorylation was found in the hepatopancreas of crab *Eriocheir sinensis* under stress [42]. These results underlined that the hepatopancreas in female shrimps mainly involved self-protection through the specific up-regulation of immunity-related genes 24 h after MF treatment. For male individuals, the specifically up-regulated DETs clustered in C2 were primarily associated with sugar and chitin metabolism. In arthropods, several classes of chitinases may have different biological functions, including the digestion of chitin-containing food, participation in molting, and involvement in immune processes [43,44,45]. The expansion of the chitinase family was discovered in *L. vannamei*, and their roles were underlined during molting [46]. In this study, multiple transcripts of chitinase were found to be significantly regulated in both sexes of *L. vannamei* after MF injection. And a greater number of differentially expressed chitinase transcripts were found in males than in females (Table S5). After MF injection, *CHIT1* mRNA in male shrimps was significantly up-regulated. The chitinase gene *CHIT1* in Decapoda is believed to be homologous to the Class I chitinase in insects [43]. RNA interference experiments with the Class I chitinase gene *TcCHT5* in the red flour beetle *Tribolium castaneum* have shown that the Class I chitinase gene plays an important role in the molting process from pupa to adult [47]. Moreover, studies in insects and decapods have shown that the expression level of Class I chitinase reaches its peak during the molting process [44,48]. These studies demonstrated the function of *CHIT1* involved in molting. In addition to *CHIT1*, 15 chitinase mRNAs were specifically up-regulated in male shrimp, and their roles need to be further elucidated.

After different concentrations of MF injection, a dose effect of MF treatment was found. Consistent with previous research, MF had a dose-dependent regulation role in the molting and growth of crustaceans [49,50]. The dietary supplementation of MF induced the molting and growth of cultured male crab *Oziothelphusa senex senex* via a dose-dependent manner [49]. In spider crab *Libinia emarginata*, MF accompanied with ecdysteroids participated in the allometric growth of claws and a high concentration had an inhibitory effect [50]. These studies mainly focused on the morphological changes, and little is known about the molecular basis of dose-dependent MF functioning. In our study, sex–dose-specific DETs clusters responding to MF1k and MF5k were identified. For the female shrimps, the specific down-regulated DETs of the low-dose group Hp_female1k were mainly enriched in “Starch and sucrose metabolism”, “Glycerophospholipid metabolism”, and the “Notch signaling pathway”, which are important pathways involved in growth and development. In freshwater prawn *Macrobrachium rosenbergii*, glycerophospholipid metabolism was also involved in growth performance [51]. The Notch signaling pathway is a highly evolutionary conserved signaling pathway, which modulates many biological processes such as cell differentiation, tissue development, and immune response [52]. Similarly in the high-dose group Hp_female5k, the DETs were also related to pathways of sugar and lipid metabolisms, such as “Amino sugar and nucleotide sugar metabolism”, “Sphingolipid metabolism”, and the “Biosynthesis of unsaturated fatty acids”. In addition, the PPAR signaling pathway was also enriched, and was found to be involved in the lipid metabolism and immune responses of crustaceans [53,54]. For the male shrimps, “Arginine and proline metabolism”, “Fatty acid biosynthesis”, “Necroptosis”, and the “p53 signaling pathway” were specifically down-regulated in the low-dose group Hp_male1k. Consistent with our results, the down-regulated expression of the p53 signaling pathway was found to mitigate the apoptosis of *L. vannamei* [55]. On the other hand, the energy metabolism-related pathway was also enriched in the high-dose group Hp_male5k, such as “Purine metabolism” and “Pyruvate metabolism”. And the same pathway of necroptosis was also presented. These results suggest that energy metabolism and immune responses were regulated differently by MF in a dose-dependent way for both sexes in shrimps.

Furthermore, through co-expression network analysis, 8 sex-specific response modules and 55 key regulatory transcripts were identified. For the female-specific module, arginine kinase (AK) was a key factor in the co-expression network. AK is a phosphotransferase that plays a critical role in energy metabolism in invertebrates [56,57]. A previous study in *L. vannamei* suggested that AK might play an important role in the coupling of energy production and utilization and the immune response in shrimps [58]. Among the key transcripts of male-specific modules, elongation of very long chain fatty acids protein 6 (*ELOV6*), tropomyosin (*TM*), and thioredoxin reductase (*TRXR*) were characterized. The high expression level of *ELOV6* in the hepatopancreas was found in other crustaceans, and its functions of energy expenditure and immune responses were also emphasized [59,60]. It could be speculated that AK and ELOV6 were the key dimorphic response genes for energy metabolism and stress resistance in their respective sex-specific modules after MF injection. In addition, the expression of *TM* was found to be correlated closely with growth patterns through the molting cycle [61]. And *TM* has also been reported to be involved in the molting process of *Penaeus monodon* [62]. TRXR is an important reductase of the antioxidant system, and studies in crab and water flea indicated the key roles of *TRXR* in immune responses [63,64]. For the sex–dose-specific modules, cysteine dioxygenase (*CDO*), lysosomal acid lipase (*LIPA*), estradiol 17-beta-dehydrogenase 8 (*HSD17B8*), and sodium/potassium-transporting ATPase subunit alpha (*ATP1A*) expressed differentially under variant MF doses suggested their vital roles in response to MF. These genes were mainly involved in amino acid and lipid metabolism. CDO is a key enzyme in taurine synthesis and plays an important role in regulating taurine content in the body [65]. Meanwhile, a study on crustacean has shown that taurine titers change periodically during the molting cycle [66]. To our knowledge, *HSD17B8* is mainly involved in lipid metabolism in mammals [67,68]. However, functional studies of *HSD17B8* in crustaceans are still lacking. LIPA can hydrolyze triglycerides, which are the main storage form of crustacean lipids, and provide the body with the necessary energy supply [69,70]. In summary, multiple genes related to amino acid, lipid, and energy metabolism were expressed in a sexually dimorphic way and were involved in growth regulation in shrimps after MF injection. Further functional experiments are needed to better understand the specific roles of them in metabolic processes and growth regulation.

## 4. Materials and Methods

### 4.1. Animal Culture

The healthy *L. vannamei* used in this experiment were purchased from a local shrimp farm in Lingshui, Hainan province, China. The average weights of the female and male shrimps were 8.58 ± 1.58 (male) and 8.37 ± 1.37 (female) g, respectively. The shrimps were divided into control and experimental groups for both sexes. Before treatment, shrimps were reared in 110 L plastic tanks with recirculating filtered artificial seawater (30‰ of salinity, 28 °C of temperature) in the Laboratory of Tropical Marine Germplasm Resources and Breeding Engineering, Sanya Oceanographic Institution, Ocean University of China. The salinity of the artificial seawater was measured by a salinity meter. Shrimps were provided with commercial food pellets (46% crude protein, 8% crude lipid, 36% carbohydrates, 10% moisture, 11% ash, 16.7 kJ/g digestible energy) twice daily with 2% of their body weight.

### 4.2. Hormone Treatment and Tissue Sample Collection

Methyl farnesoate (Echelon Biosciences, Salt Lake City, UT, USA) was dissolved in ethanol as stock solution (1 mg/mL) for further usage. PBS buffer was adopted as a diluent for the preparation of the MF injection solution. Each individual was injected in the second lateral muscle by a 1 mL syringe with a 0.45 mm needle. The control groups were injected with 60 μL of PBS, while the two experimental groups were injected with 1000 ng (MF1k) and 5000 ng of MF (MF5k) in 60 μL of PBS, respectively. According to previous studies in crustaceans [13,71], at 24 h post injection, the hepatopancreases were dissected and collected, which were frozen in liquid nitrogen immediately and then stored at −80 °C until use. For RNA sequencing, the total RNA of each tissue was isolated using TRIzol reagent (Invitrogen™, Thermo Scientific, Waltham, MA, USA) as per the manufacturer’s instruction. The quantity and quality of the extracted total RNA were evaluated using a Nanodrop spectrophotometer (NanoDrop one, Thermo Scientific, Waltham, MA, USA) and gel electrophoresis (Mini-SubCellGT, Bio-Rad, Hercules, CA, USA).

### 4.3. RNA Sequencing and Data Processing

To obtain the expression profile of both sex shrimps responding to MF injection, the RNA samples of three individuals from each group were extracted. In total, 18 RNA samples from the hepatopancreases of shrimps were subjected to Illumina transcriptome library construction and sequencing at BGI Company (Sanya, China).

The raw data from Illumina sequencing were trimmed and quality filtered via a combination of Cutadapt v4.1 and Trimmomatic v0.36 software. To obtain the transcripts’ expression profile of 18 samples, the filtered clean reads from the Illumina sequencing data were mapped to the assembled transcripts’ reference [46] with Bowtie2 v2.2.5. The mapping results were then converted to count matrix by RSEM [72] and the expression profiles of mRNA were determined by TPM (transcripts per million). Transcripts were determined to be expressed only if they had a TPM > 0.1 in at least two of the three replicates.

### 4.4. Differential Expression and Functional Analyses

Differential expression analysis was conducted with the edgeR v3.28.1 package and significantly differential expression was defined as *p*-value < 0.05 and |log2FoldChange (FC)| > 1.5. The clustered profile of differentially expressed transcripts (DETs) determined by Log2FC values was visualized with R package ClusterGVis v0.1.1 [27]. KEGG and GO enrichment analysis of DETs was performed using the clusterProfile package, and visualized using the ggplot2 package in R.

The weighted gene co-expression network analysis (WGCNA) was performed with the filtered expression profile from hepatopancreases using the R package WGCNA [73]. Function-annotated transcripts with the top 25% variance were chosen for the network analysis. Sex-specific modules were determined via overrepresentation analysis of clustered DETs in modules using a hypergeometric test, and modules with *p*-value < 0.01 were considered as the sex-specific modules. The top 50 hub transcripts of a given module were identified based on the intramodular connectivity values. The hub transcripts appearing in both sex-specific modules and corresponding DET clusters were considered to be the key transcripts.

## 5. Conclusions

In conclusion, this study comprehensively explored the dimorphic responses of the hepatopancreas in female and male *L. vannamei* after MF injection. By comparative analysis of transcriptome, sex-specific and sex–dose-specific response DET sets were characterized, as well as key co-expression modules and regulatory transcripts. Functional analysis of DETs showed that the male-specific DETs were mainly related to sugar and lipid metabolism, of which multiple chitinases were significantly up-regulated. In contrast, the female-specific DETs were mainly related to miRNA processing and immune responses. For the sex-specific modules, the key transcripts of genes related to energy metabolism and immune responses were identified. Taken together, our findings provide new insights into the MF regulation and advance the understanding of the molecular basis behind sexual dimorphism in shrimp.

## Data Availability

The datasets supporting this article are included within the article and its supplementary information. Other details will be made available on reasonable request.

## Supplementary Materials

The following supporting information can be downloaded at: https://www.mdpi.com/article/10.3390/ijms25158152/s1.

## Abbreviations

MF	methyl farnesoate
JH	juvenile hormone
Hp	hepatopancreas
TPM	transcripts per million
DEGs	differentially expressed genes
DETs	differentially expressed transcripts
WGCNA	weighted gene co-expression network analysis
FC	FoldChange
JHE-L	juvenile hormone esterase-like carboxylesterase
Vg	vitellogenin
VgR	vitellogenin receptor
DDX17	DEAD-box helicase 17
SMAD3	mothers against decapentaplegic homolog 3
AK	arginine kinase
ELOV6	very long chain fatty acids protein 6
TM	tropomyosin
TRXR	thioredoxin reductase
CDO	cysteine dioxygenase
LIPA	lysosomal acid lipase
HSD17B8	estradiol 17-beta-dehydrogenase 8
ATP1A	sodium/potassium-transporting ATPase subunit alpha
