# Supplementary material for: Comparative Transcriptome Analysis of Hepatopancreas Reveals Sexual Dimorphic Response to Methyl Farnesoate Injection in Litopenaeus vannamei
Source: Int J Mol Sci. 2024 Jul 26;25(15):8152. doi: 10.3390/ijms25158152 (PMC11311334; doi:10.3390/ijms25158152)
Supplement: Supplementary file 1 [file ijms-25-08152-s001.zip › Supplementary Figures.docx]

**Supplementary Materials**

Table S1. Summary of Illumina RNA-Seq data.

Table S2. Information of transcript variants in the key pathways related to hormone.

Table S3. Information of the gender specific responding DETs clusters.

Table S4. Enriched GO terms of DETs in each sex-specific cluster.

Table S5. Male-specific up-regulated transcripts of chitinase in C2 cluster.

Table S6. The key transcripts identified based on sex-specific DETs and modules.

Figure S1. Statistics of DETs in hepatopancreases of MF-injection groups compared to their control group.

Figure S2. Overview of the WGCNA construction.

Figure S3. Overrepresentation analysis of the sex-specific DETs in co-expression modules.


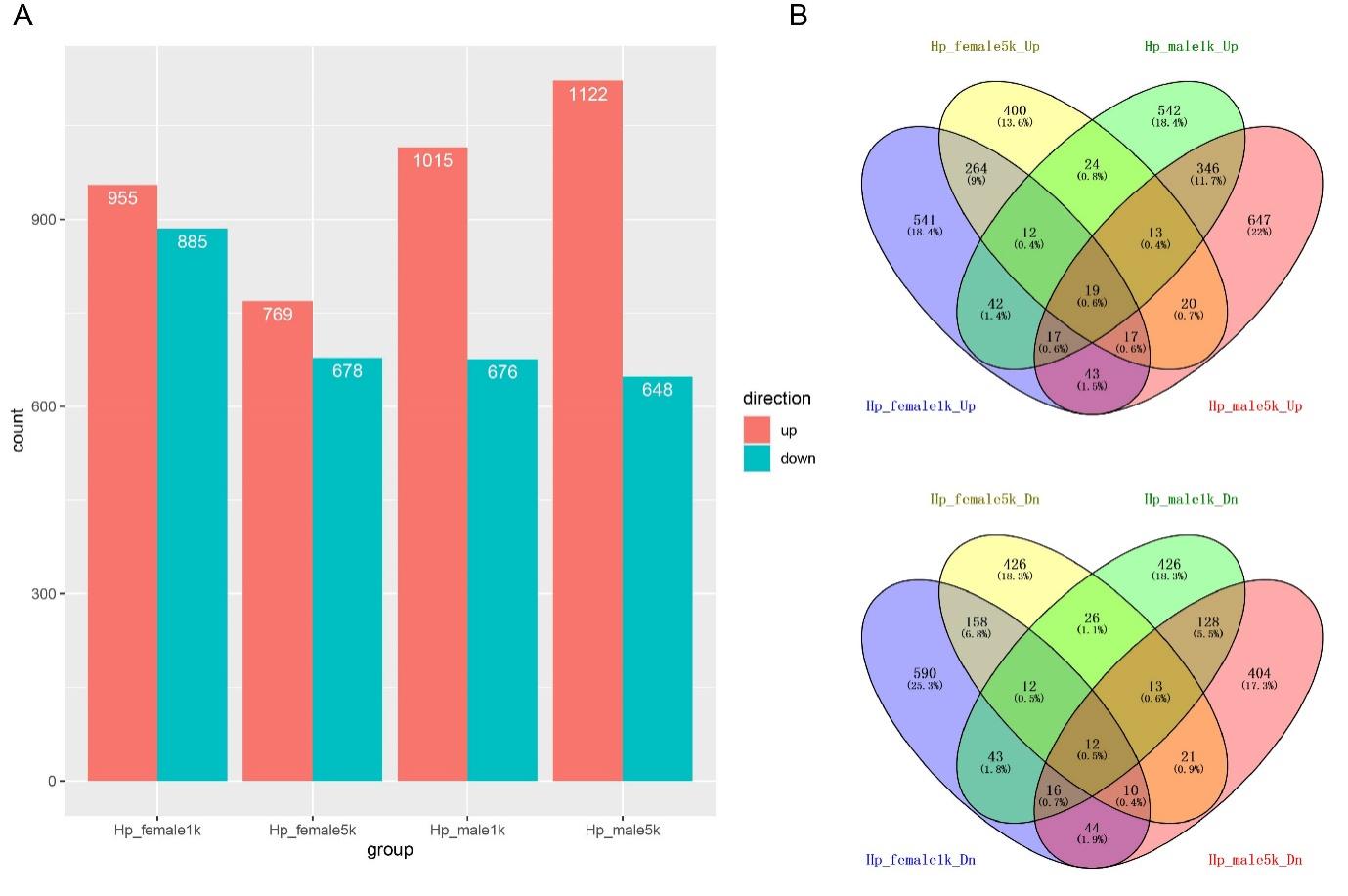


**Fig. S1** Statistics of DETs in hepatopancreases of MF-injection groups compared to their control group. (A) Histogram of DETs number in each experimental group. (B) Venn plot showing the intersections of upregulated and downregulated DETs in four groups. Hp, hepatopancreases; female1k, female5k, male1k and male5k represent female and male shrimps injected with 1000 and 5000 ng MF, respectively.


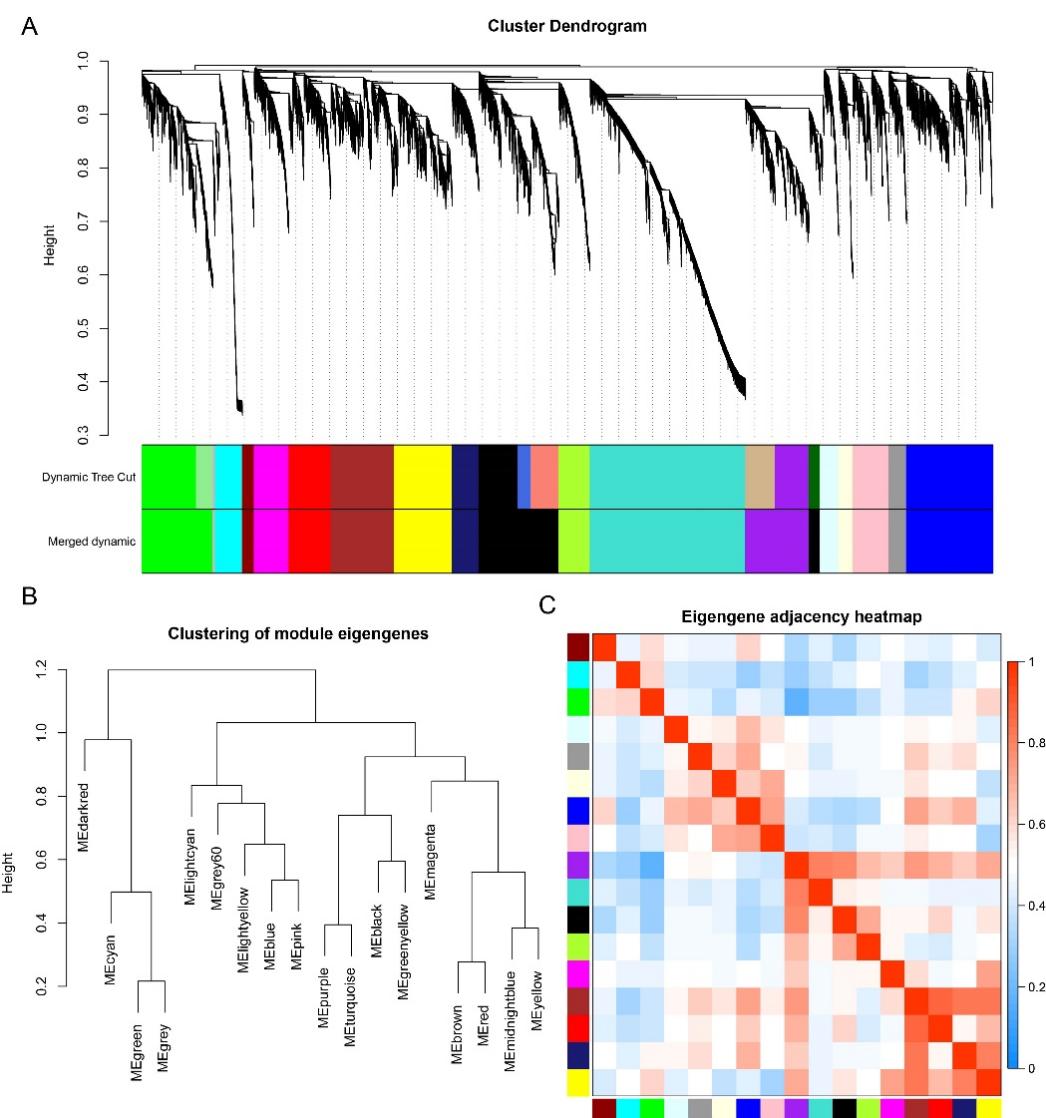


**Fig. S2** Overview of the WGCNA construction. (A) Clustering dendrogram of genes, with dissimilarity based on topological overlap, together with assigned module colors. (B) Clustering of module eigengenes. (C) Adjacency heatmap of module eigengenes. Red and blue indicate the high and low correlation, respectively.


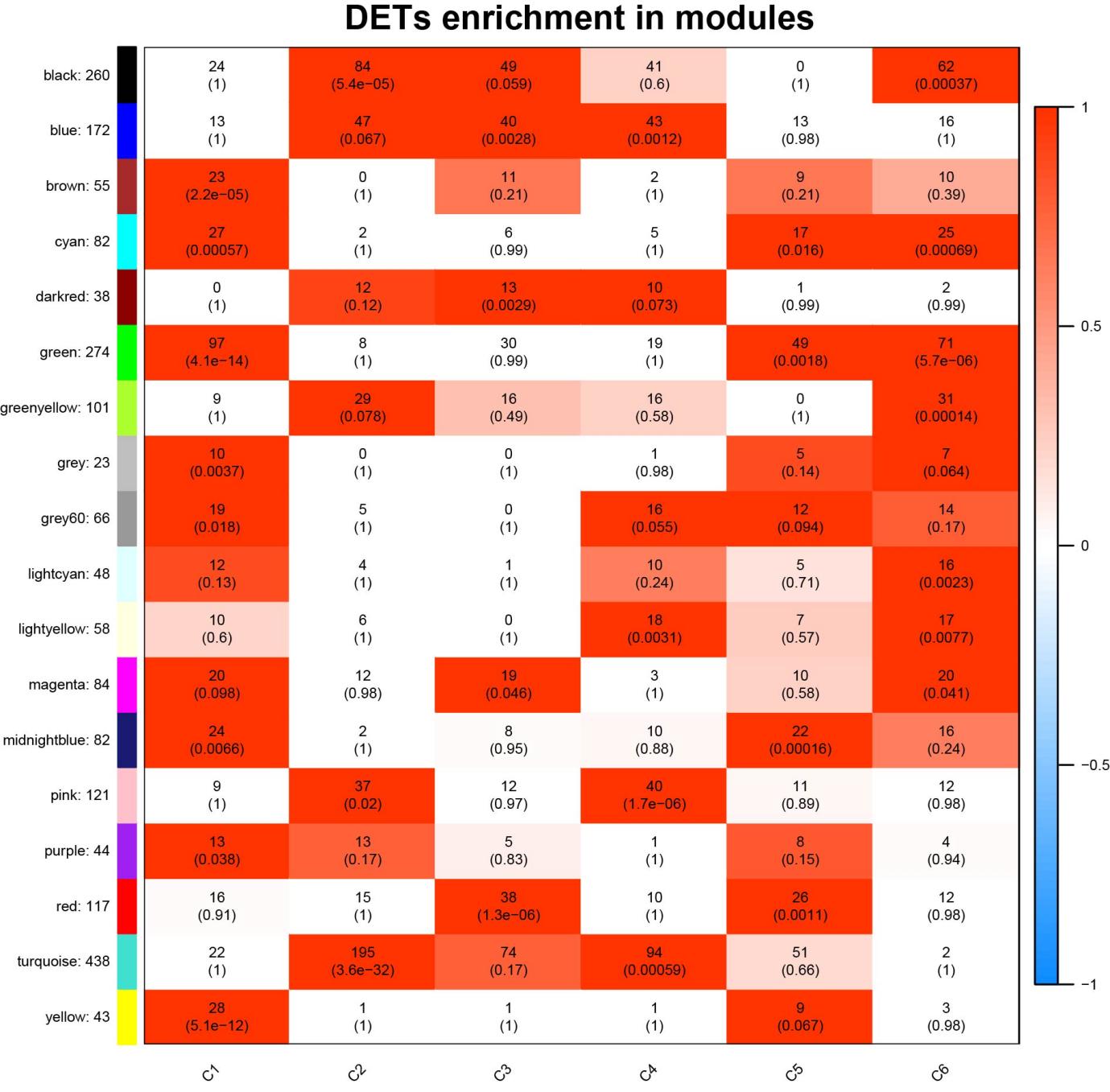


**Fig. S3** Overrepresentation analysis of the sex-specific DETs in co-expression modules. The upper numbers in the grid represent the number of clustered DETs enriched in modules. The number below in the grid represent the enrichment significance (*p*-value).
